# A Posteriori Error Analysis for a Coupled Stokes-Poroelastic System with Multiple Compartments

**DOI:** 10.1007/s10915-025-02814-3

**Published:** 2025-03-08

**Authors:** Ivan Fumagalli, Nicola Parolini, Marco Verani

**Affiliations:** https://ror.org/01nffqt88 grid.4643.5 0000 0004 1937 0327 MOX, Dipartimento di Matematica, Politecnico di Milano, Piazza Leonardo da Vinci 32, 20133 Milan, Italy

**Keywords:** Fluid-poromechanics interaction, Multiple-network poroelasticity, A posteriori estimates, Cerebrospinal fluid, 65N12, 65N22, 65N30, 76Z05

## Abstract

The computational effort entailed in the discretization of fluid-poromechanics systems is typically highly demanding. This is particularly true for models of multiphysics flows in the brain, due to the geometrical complexity of the cerebral anatomy—requiring a very fine computational mesh for finite element discretization—and to the high number of variables involved. Indeed, this kind of problems can be modeled by a coupled system encompassing the Stokes equations for the cerebrospinal fluid in the brain ventricles and Multiple-network Poro-Elasticity (MPE) equations describing the brain tissue, the interstitial fluid, and the blood vascular networks at different space scales. The present work aims to rigorously derive a posteriori error estimates for the coupled Stokes-MPE problem, as a first step towards the design of adaptive refinement strategies or reduced order models to decrease the computational demand of the problem. Through numerical experiments, we verify the reliability and optimal efficiency of the proposed a posteriori estimator and identify the role of the different solution variables in its composition.

## Introduction

The numerical modeling of multiphysics flows in the human brain poses several difficulties, due to the complexity of the brain’s geometry and the computational cost of handling several coupled physical systems. This modeling is of paramount importance in the investigation of the Cerebrospinal Fluid (CSF), whose main functions are to wash out the waste products of cerebral activity and protect the brain from impact with the skull [23, 35]. The CSF is generated in the cerebral tissue by mass exchange through the walls of capillary blood vessels and permeates the whole organ in its interstitial space: the interaction between these fluid networks and the elastic tissue can be modeled by Multiple-network Poro-Elasticity (MPE) equations [14, 15, 22, 27]. This system is then coupled with the CSF flowing in the hollow cavities of the cerebral ventricles and the subarachnoid spaces, where the CSF flow can be modeled by Stokes equations [19, 20, 29].

The large number of variables encompassed by fluid-poromechanics models and the geometrical complexity of the brain and fluid-tissue interface make the numerical simulation of the problem particularly demanding. To reduce the computational effort, different strategies can be considered: adaptive refinement allows for retaining geometric accuracy while decreasing the computational demands, while reduced-order models provide an efficient means to approximate the solution of complex problems for different values of the model parameters [25, 32, 33, 38]. Both these strategies are classically based on a posteriori error estimates, which have been derived for several single-physics problems, including single-fluid Biot equations [2, 28, 34] or (Navier-)Stokes equations [3, 8, 24, 37]. An a posteriori analysis of coupled Biot-Stokes system has been carried out in [6, 26, 39], but the case of multiple interacting fluids is scarcely covered by the a posteriori literature: the MPE problem alone has been addressed only in [30] for a particular case and in the recent work [16], but the coupled MPE-Stokes problem with time-dependent pressure equations seems to be missing.

The present work aims at filling this gap, providing rigorous a posteriori estimates for the coupled MPE-Stokes problem with time-dependent pressure equations. Specifically, we provide reliable residual-based estimators in the abstract framework of [17], enhanced to account for multi-domain problems. Moreover, through numerical experiments, we analyze the efficiency of these estimators and assess their main components.

Starting from Sect. 2, we introduce the MPE-Stokes problem and its discretization by finite elements in space and the implicit Euler scheme in time. Then, Sect. 3 is devoted to the derivation of a posteriori error estimates for the solution to the problem. In Sect. 4 we analyze the reliability and efficiency of the estimators and discuss the relevance of their main components.

## The Coupled Stokes-MPE System

**Fig. 1:**
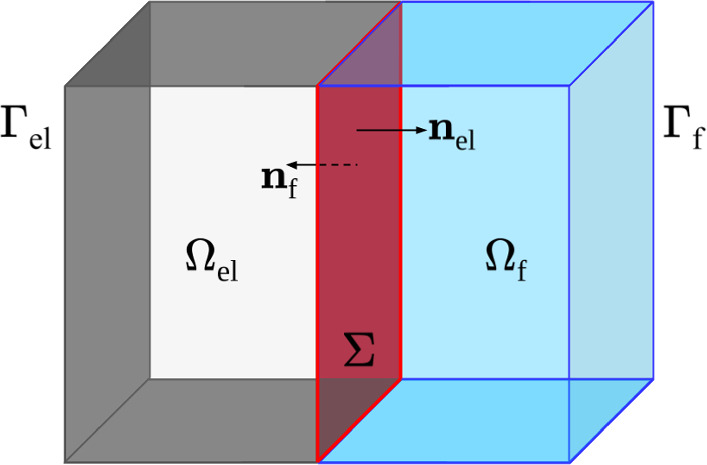
Domain scheme: poroelastic domain $\varOmega _\textrm{el}$ (light grey), Stokes’ domain $\varOmega _\textrm{f}$ (blue), interface $\varSigma$ (red), and external boundaries $\varGamma _\text {el}=\varGamma _{\text{ D },\varvec{d}}\cup \varGamma _{\text{ N },\varvec{d}}=\varGamma _{\text{ D },J}\cup \varGamma _{\text{ N },J}$

Let us consider a polyhedral ${\mathfrak {D}}$-dimensional domain $\varOmega \subset {\mathbb {R}}^{\mathfrak {D}}$ (${\mathfrak {D}}=2,3$), schematically represented in Fig. 1, partitioned into a poroelastic region $\varOmega _\textrm{el}$ and a fluid region $\varOmega _\textrm{f}$ by an interface $\varSigma =\partial \varOmega _\textrm{el}\cap \partial \varOmega _\textrm{f}$ composed of a finite number of flat polygons. Stokes equations are set in $\varOmega _\textrm{f}$, with $\varvec{u}$ and *p* denoting the fluid’s velocity and pressure, while $\varOmega _\textrm{el}$ is filled with a linear poroelastic medium subject to the MPE equations, with solid displacement $\varvec{d}$ and network pressures $p_\textrm{j}\in J$, where *J* is a set of $\#J$ indices. The external boundaries $\varGamma _\textrm{el}=\partial \varOmega _\textrm{el}\setminus \varSigma , \varGamma _\textrm{f}=\partial \varOmega _\textrm{f}\setminus \varSigma$ are partitioned in Dirichlet and Neumann portions for the different variables, with clear notation: $\varGamma _\text {el}= \varGamma _{\text{ D },\varvec{d}}\cup \varGamma _{\text{ N },\varvec{d}}=\varGamma _{\text{ D },J}\cup \varGamma _{\text{ N },J}, \varGamma _\text {f}=\varGamma _{\text{ D },\varvec{u}}\cup \varGamma _{\text{ N },\varvec{u}}$; notice that, for simplicity, we consider the same Dirichlet/Neumann splitting of the boundaries for all fluid networks $\textrm{j}\in J$. We consider the Stokes and linear elasticity equations to be steady, while the time dependence of the porous flow is accounted for in the porous fluid momentum equations, as follows: 
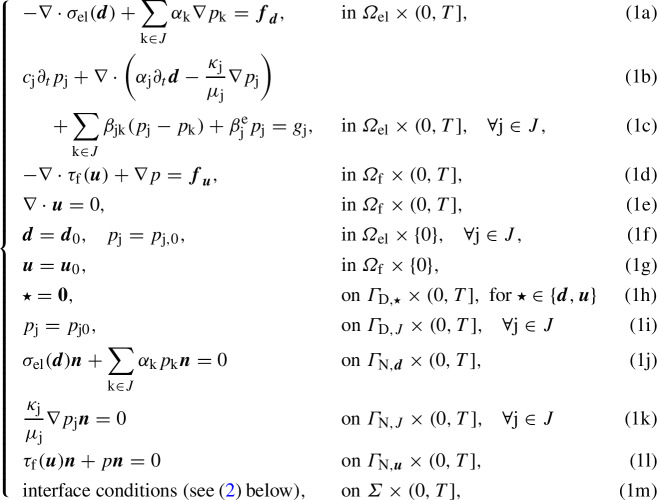


where  is the Cauchy stress tensor of the elastic medium and  is the viscous stress tensor of the fluid – with . We assume that, among all the fluid networks indexed in *J*, only one exchanges mass at the interface $\varSigma$ with the Stokes domain  and we denote it by $\textrm{E}$: the others exchange mass only among themselves and with  (cf. the terms with  in (1c) and Remark 2.1 below). Accordingly, the following interface conditions are imposed on $\varSigma$, for all times $t\in (0,T]$ (with an indication of the physical meaning of each condition):
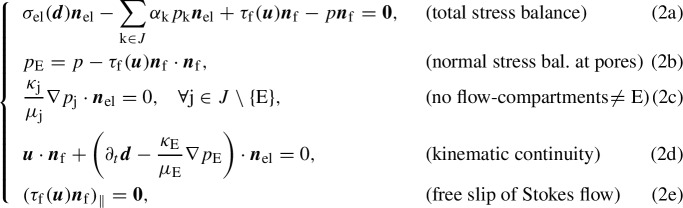


where $(\varvec{\phi })_\parallel =\varvec{\phi }-(\varvec{\phi }\cdot \varvec{n}_\text {f})\varvec{n}_\text {f}$ is the tangential component of $\varvec{\phi }$ along $\varSigma$.

### Remark 2.1

*(Specifics of brain modeling)* In the case of brain multiphysics flow, the fluid networks index set $J=\{\text {A},\text {V},\text {C},\textrm{E}\}$ contains the arterial (A), venous (V) and capillary (C) blood flow and the extracellular/interstitial cerebrospinal fluid ($\textrm{E}$). In this framework:

- conditions (2c) prevent flow of the blood compartments A, V, C through the interface $\varSigma$, consistently with the physiological brain-blood barrier [1];
- the kinematic condition (2d) enforces mass conservation by balancing the CSF flow between the interstitial space (in $\varOmega _\textrm{el}$) and the brain ventricles (in $\varOmega _\textrm{f}$), net of the normal interface velocity $\partial _t\varvec{d}\cdot \varvec{n}_\text {el}$ due to tissue displacement;
- stress balance across the interface as a whole is ensured by (2a), while at the interface pores we consider the free-slip conditions (2b)-(2e).

Regarding the elasticity and Stokes Eqs.(1a)-(1d), we consider a quasi-static approximation. This is a viable option when we can assume that the load on the elastic structure and on the fluid domain, induced by the dynamics of the fluid in the porous compartments, has a time scale that is much larger than the characteristic times of the elastic structure and of the Stokes flow. In brain physiology, this is particularly relevant in the study of hydrocephalus development. This pathology is characterized by an over-production of CSF (modeled by the unsteady Eq. (1c)), but it develops in a time scale of days/weeks [36]: this is significantly longer than the heartbeat and respiration period, which are the time scales at which tissue displacement and CSF pulsatility are relevant [7, 20, 40]. Therefore we can assume negligible inertial effects for the tissue and for the CSF in the brain ventricles.

### Variational Formulation

We introduce the following Sobolev spaces, with $\textrm{j}\in J$:

3 $$\begin{aligned} \begin{aligned} {V_{\text {j}}}&{=} H^1_{\varGamma _{\text{ D },J}}(\varOmega _\text {el})&{V_{\varvec{d}}}&{=} [H^1_{\varGamma _{\text{ D },\varvec{d}}}(\varOmega _\text {el})]^{\mathfrak {D}},&{V_{\varvec{u}}}&{=} [H^1_{\varGamma _{\text{ D },\varvec{u}}}(\varOmega _\text {f})]^{\mathfrak {D}}, \\ {L_{\text {j}}}&{=} L^2(\varOmega _\text {el}),&{L_{\varvec{d}}}&{=} [L^2(\varOmega _\text {el})]^{\mathfrak {D}},&{L_{\varvec{u}}}&{=} [L^2(\varOmega _\text {f})]^{\mathfrak {D}},\\ {V_J}&{=} [{V_{\text {j}}}]^{\#J},&{L_J}&{=}[{L_{\text {j}}}]^{\#J},&{L_p}&{=} L^2(\varOmega _\text {f}). \end{aligned} \end{aligned}$$

To simplify the notation, we employ $\varvec{p}_J=[p_\text {j}]_{\text {j}\in J}$ to indicate the elements of ${V_J}$ and ${L_J}$: these functions are vector fields of dimension $\#J$ having $p_\textrm{E}$ as their last component. To account for the time dependence of the system variables, we set up our problem in the Bochner spaces , where ${\mathscr {V}}$ is any Hilbert space introduced in (3). With analogous notation, we will also consider the spaces .

The variational formulation of problem (1) reads as follows:

Find  such that, given $\varvec{d}|_{t=0}=\varvec{d}_0$ $\varvec{p}_J|_{t=0}=[p_{\text {j},0}]_{\text {j}\in J}$ in $\varOmega _\textrm{el}$ and $\varvec{u}|_{t=0}=\varvec{u}_0$ in $\varOmega _\textrm{f}$, the following equations hold for a.e. $t\in [0,T]$, 
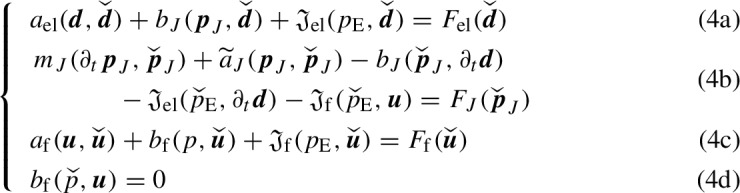


for all , whereIn these definitions, $(\cdot ,\cdot )_D$ denotes the $L^2$ product over a domain *D*. Analogously, $\Vert \cdot \Vert _D$ will denote the norm of $L^2(D)$.

### Time and Space Discretization

We introduce a simplicial mesh ${\mathscr {T}}$ partitioning the whole domain $\varOmega$, and we split it into two submeshes ${\mathscr {T}}_\textrm{el},{\mathscr {T}}_\textrm{f}$, corresponding to the subdomains $\varOmega _\textrm{el},\varOmega _\textrm{f}$ and conforming with the interface $\varSigma$. The sets of codimension-1 internal facets are denoted by ${\mathscr {F}}_\textrm{el}^I,{\mathscr {F}}_\textrm{f}^I$ (triangles for ${\mathfrak {D}}=3$, line segments for ${\mathfrak {D}}=2$). Analogously, we denote by ${\mathscr {F}}_\varSigma$ the facets lying on the interface $\varSigma$ and by ${\mathscr {F}}_{\text {D},\phi }$ the facets lying on the corresponding Dirichlet boundary $\varGamma _{\text{ D },\phi }, \phi \in \{\varvec{d},\varvec{p}_J,\varvec{u}\}$. In the following, we will denote by $h_{K}$ the characteristic size of an element ${K}\in {\mathscr {T}}$.

On this partitioning, we introduce the following conforming finite element spaces:

5 $$\begin{aligned} \begin{aligned} X_\star ^r=\{\phi _h\in C^0({\overline{\varOmega }}_\star ) :\phi _h|_{K}\in {\mathbb {P}}^r({K})\ \forall {K}\in {\mathscr {T}}\}, \quad \star \in \{\text {el},\text {f}\},\\ V_{\varvec{d},h}= {V_{\varvec{d}}}\cap [X_\text {el}^{s+1}]^{\mathfrak {D}}, \qquad V_{J,h}= {V_J}\cap [X_\text {el}^{s+1}]^{\#J}, \\ L_{\varvec{d},h}= {L_{\varvec{d}}}\cap [X_\text {el}^{s+1}]^{\mathfrak {D}}, \qquad L_{J,h}= {L_J}\cap [X_\text {el}^{s+1}]^{\#J}, \\ V_{\varvec{u},h}= {V_{\varvec{u}}}\cap [X_\text {el}^{s+1}]^{\mathfrak {D}}, \qquad L_{\varvec{u},h}= {L_{\varvec{u}}}\cap [X_\text {el}^{s+1}]^{\mathfrak {D}}, \qquad L_{p,h}= {L_p}\cap X_\text {el}^s. \end{aligned} \end{aligned}$$

We denote by  suitable interpolation operators onto the discrete spaces introduced above, such that the following interpolation estimate holds:6and analogous ones for . For example, Clément interpolators can be employed [17]. We anticipate that no interpolation operator is needed over the Stokes pressure space ${L_p}$.

Regarding time discretization, we consider an implicit Euler scheme on a uniform time grid made of $N_T$ subintervals  of length $\varDelta t$, with $t^0=0, N_T=T/{\varDelta t}$. All the a posteriori results presented hereafter can be easily extended to nonuniform time discretization, as done, e.g., in [16, 17, 37].

In the following, for any function  we will use the notation  to indicate its full space-time discretization evaluated at time , whereas  will denote the continuous piecewise linear (in time) function such that . We also introduce the projector $\pi ^0$ onto piecewise constant functions in time, such that .

Therefore, the fully discrete problem approximating (4) reads as follows:

Find , piecewise linear in time, such that, for a.e. $t\in [0,T]$, 
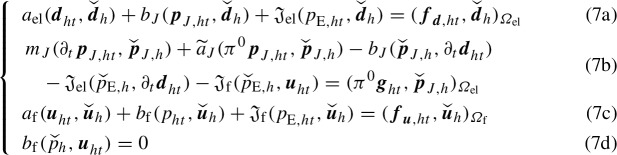
 for all , and . Notice that  and  a.e. in (0, *T*), where

## *A Posteriori* Error Analysis

Throughout this section, the notation $a\lesssim b$ means that there exists a constant $C>0$, independent of discretization parameters (but possibly dependent on data and on the final time *T*), such that $a\leqslant Cb$.

The a posteriori analysis that we present is based on the following properties of the continuous problem (4), inspired by the abstract framework of [17]:

### Proposition 3.1

Under the definitions of Sect. 2.1, assuming that and that each of the Dirichlet boundaries $\varGamma _{\text{ D },\star }, \star =\varvec{d},\varvec{u},p_\text {j}, \forall \text {j}\in J,$ is not empty, the following properties hold:

1. The forms $a_\text {el}:{V_{\varvec{d}}}\times {V_{\varvec{d}}}\rightarrow {\mathbb {R}}$, ${\widetilde{a}}_J:{V_J}\times {V_J}\rightarrow {\mathbb {R}}$, $a_\text {el}:{V_{\varvec{d}}}\times {V_{\varvec{d}}}\rightarrow {\mathbb {R}}$, $m_J:{L_J}\times {L_J}\rightarrow {\mathbb {R}}$ are bilinear, symmetric, coercive, and continuous in their spaces of definition.
2. The norms induced by these forms fulfill the following inequalities for all :
3. The Stokes operator  satisfies the following inf-sup inequality:  s.t.

Therefore, we can consider the spaces ${V_{\vec {d}}},{V_J},{L_J},{V_{\vec {u}}}$ as endowed with the norms induced by $a_\textrm{el},{\widetilde{a}}_J,m_J,a_\textrm{f}$, respectively. Moreover, the properties above also hold at the discrete level, with a discrete inf-sup constant  in point 3.

### Proof

The proof of points 1-2 is straightforwardly based on the definition of coercivity and continuity, and it exploits Korn/Poincaré inequalities. Point 3 is discussed in classical works on Stokes equations, e.g. [11, 21]. $\square$

Based on the properties of Proposition 3.1, we can prove the following result:

### Lemma 3.1

The discrete problem (7) is well-posed.

### Proof

The proof is inspired by an analogous result of [17] for an elliptic-parabolic problem. We consider problem (7) at time $t=t^n$ and choose the following test functions: . Summing up all the equations of the problem and using that $a_\text {el}(\varvec{d}_1,\varvec{d}_1-\varvec{d}_2)=\frac{1}{2}a_\text {el}(\varvec{d}_1,\varvec{d}_1)+\frac{1}{2}a_\text {el}(\varvec{d}_1-\varvec{d}_2,\varvec{d}_1-\varvec{d}_2)-\frac{1}{2}a_\text {el}(\varvec{d}_2,\varvec{d}_2)$ for any $\varvec{d}_1,\varvec{d}_2\in {V_{\varvec{d}}}$ – and analogous identities for the other bilinear forms – yieldswhere all coupling terms involving forms $b_J,b_\textrm{f},{\mathfrak {J}}_\textrm{el},{\mathfrak {J}}_\textrm{f}$ cancel out due to the block-skew-symmetry of the problem. By Young and Cauchy–Schwarz inequalities, and thanks to properties 1-2 of Proposition 3.1, this implies the following inequality:This stability estimate, combined with the inf-sup inequality of Proposition 3.1 (property 3), ensures the uniqueness of the solution at time  in (7). Since the fully discrete problem can be reformulated as a square linear system for the degrees of freedom of the discrete variables, uniqueness is enough to prove well-posedness, thus the proof is concluded. $\square$

We now introduce the following consistency operators, namely the residuals of problem (7) tested against continuous test functions:8and by the usual notation, we denote by  their evaluation at time .

We are now ready to introduce a preliminary result estimating the discretization errors in terms of the consistency operators.

### Theorem 3.1

Let the errors at each time *t* be denoted as (the latter including ). Then, under the assumptions of Proposition 3.1, the following estimate holds:9where10

### Proof

Let us also introduce  at each time *t* (including its component ). Subtracting the discrete problem (7) from the continuous problem (4), both tested against generic continuous test functions , yields the following error problem, holding for a.e. $t\in [0,T]$:11We now choose  as test functions and we sum up the equations in (11), noticing that the terms involving the forms $b_J, b_\textrm{f}, {\mathfrak {J}}_\textrm{el}, {\mathfrak {J}}_\textrm{f}$ cancel out for this choice of the test functions. Moreover, we observe that the following equalities hold:due to the symmetry of the forms and the definition of . Therefore, we obtain the following identity:12We integrate (12) in time from 0 to a generic $\overline{t}$ and we use the following integration-by-parts formula:Then, using the coercivity of the forms $a_\textrm{el},{\widetilde{a}}_J,m_J,a_\textrm{f}$ (see Proposition 3.1) and employing the Cauchy–Schwarz and the Young inequalities yield the following inequality:Using the Cauchy–Schwarz and the Young inequalities also in the time integrals and in the dualities in the spaces ${V_{\varvec{d}}},{V_{\varvec{u}}},{L_p},{V_J}$ yields13where  has been removed since it is non-negative. Now, since (13) holds for any $\overline{t}\in (0,T]$, we can take the supremum w.r.t. $\overline{t}$ over  at both sides, use Young inequality on the terms still involving , and recognize the terms defining  and . This completes the proof. $\square$

We remark that in the preliminary estimate of Theorem 3.1, the estimator  is not computable a posteriori as it depends on the errors. In the following section, we thus address its estimation in terms of local residuals and jumps. To this aim, we introduce the jump $[\![\varvec{\phi }]\!]_{F}$ of a generic vector field $\varvec{\phi }:\varOmega \rightarrow {\mathbb {R}}^{\mathfrak {D}}$ across a face ${F}$, defined as follows:

$$\begin{aligned} [\![ \varvec{\phi }]\!]_{F}= \varvec{\phi }^+\odot \varvec{n}_{F}^+ + \varvec{\phi }^-\odot \varvec{n}_{F}^- \qquad \text{ with }\qquad \varvec{\phi }\odot \varvec{n} = \frac{1}{2}(\varvec{\phi }\otimes \varvec{n}+\varvec{n}\otimes \varvec{\phi }), \end{aligned}$$

where the superscripts $+$ and − denote the restriction on either side of the face ${F}$. This jump operator is employed in the Discontinuous Galerkin community (see, e.g., [5, 19]), yet all the results still hold if the non-symmetric outer product $\varvec{\phi }\otimes \varvec{n}$ is used in place of $\varvec{\phi }\odot \varvec{n}$.

### A posteriori Estimates of the Residual Operators

We now address the estimation of the terms of Theorem 3.1 that involve the residual operators ${\mathcal {G}}_\star , \star =\varvec{d},\varvec{u},J,p$.

#### Lemma 3.2

Under the same assumptions of Theorem 3.1, the following estimates hold:

      where
      where
      where
      where

#### Remark 3.1

We point out that the local residual estimators  are the local residuals of the bulk equations (1a)-(1c)-(1d) in strong form, while the terms  are arise from combinations of the interface conditions (as highlighted by the arrows):

#### Proof

(of Lemma 3.2) We first observe that the following holds:14Denoting by  the evaluation of ${\mathcal {G}}_\star$ at time , for each of the points 1-4, we first estimate the operator norm  a.e. in time and then we wrap it into the Bochner space norm appearing in the thesis.

1. We start by estimating the numerator of 15 By the definition of ${\mathcal {G}}_{\varvec{d}}$ and element-wise integration by parts (i.b.p.), we obtain 16 where the terms with  vanish because $V_{J,h}\subset [C^0(\varOmega _\textrm{el})]^{\#J}$. Using the Cauchy–Schwarz inequality on each term and multiplying/dividing by appropriate powers of $h_{K}$ yield  Hinging upon the discrete Cauchy–Schwarz inequality applied to the sums over the elements, as well as the inequality $(a+b)^2<3(a^2+b^2)$, we can prove that 17 Then, using interpolation estimates (6) yields 18 Now, using (15), we obtain , which yields point 1 of the thesis after taking the supremum w.r.t. time at both members and noticing that each  is constant over .
2. Being ${\mathcal {G}}_{\varvec{d}}$ piecewise linear in time,  is constant on each time interval . Employing twice equality (16), for , yields 19 Then, proceeding in the same way as in point 1 of this proof, we can obtain  from which summing over all the intervals  yields
3. Recalling that, for each function  that is piecewise linear in time,
  and , we can proceed as in point 1 of this proof and show that 20 where  Then, we employ the definition of  on the left-hand side of (20) and the interpolation estimates (6) together with the Cauchy–Schwarz inequality on the right-hand side, thus obtaining . Again, the second member of this inequality is piecewise constant in time, therefore we can obtain the desired result.
4. Proceeding as in point 1, by definition (8) of ${\mathcal {G}}_{\varvec{u}}$ and ${\mathcal {G}}_p$, and using the continuous problem (4c)-(4d), we have 21 where  vanishes because $L_{p,h}\subset C^0(\varOmega _\textrm{f})$. Now, as in point 1, we use the definition of  on the left-hand side of (21), we apply integral and discrete Cauchy–Schwarz inequalities to the right-hand side, as well as the interpolation estimates for the terms tested against , thus obtaining  Therefore,  and we conclude the proof by integrating both sides of the inequality w.r.t. time and noticing that the right-hand side is piecewise constant.

$$\square $$

### A posteriori Error Estimates

We are now ready to combine the results of the previous sections to prove the following a posteriori error estimate:

#### Theorem 3.2

Under the assumptions of Theorem 3.1, the following a posteriori error estimates hold:22where

#### Proof

We proceed taking inspiration from [16, 17, 37], in which inequalities like the one in Theorem 3.1 are combined with a posteriori estimates of the consistency operators like those of Lemma 3.2. We start observing that Theorem 3.1 impliesMoreover, a combination of Lemma 3.2 and the Young inequality yields . To estimate the terms involving  and , we rely on the following error equations in operator form (holding a.e. in time), that can be obtained by a subtraction of the discrete problem (7) from the continuous one (4): 23a23b23c23d Now, computing the $L^2$ norm in time of both members of (23b)-(23c)-(23d) and applying Lemma 3.2, as well as recalling the definition (10) of , we can prove the following estimates:Finally, to estimate , we apply the time derivative $\partial _t$ to both sides of (23a), we compute the Bochner $L^1$-norm, and we apply again Lemma 3.2:This completes the proof. $\square$

To close this section, we point out that Theorem 3.2 proves the reliability of the estimator. To discuss its efficiency, one could proceed similarly to [17, Theorem 4.2] and derive upper bounds of the estimator in terms of the error. In this work, instead, we decide to deputize the assessment of the estimator’s efficiency to the numerical tests reported in the following section.

## Numerical Results

Bearing in mind the application that motivates the present work, namely brain multiphysics flow modeling, the geometrical complexity of the domain asks for adaptive mesh refinement strategies, whereas the benefit of adaptivity in time would be limited by the relatively slow and regular flow regime [7, 14, 22]. For this reason, in this section we verify the estimates of Theorem 3.2 and assess the efficiency of the estimators with respect to *h* refinement, whereas $\varDelta t$ is chosen sufficiently small not to hinder the convergence order.

We consider problem (1) in ${\mathfrak {D}}=2$ dimensions with 1 compartment $J=\{\textrm{E}\}$. In the domain $\varOmega =\varOmega _\textrm{el}\cup \varOmega _\textrm{f}=(-0.5,0)\times (0,0.5)\cup (0,0.5)\times (0,0.5)$, with interface $\varSigma =\{0\}\times (0,0.5)$, the following is a solution of (1) for proper values of the source functions $\varvec{f}_{\varvec{d}}, g_\text {E},\varvec{f}_{\varvec{u}}$:

$$\begin{aligned} \varvec{d}(t,\varvec{x})&= \left( \cos (\eta t)-\sin (\eta t)\right) \pi \frac{\kappa }{\eta } \left( \sin (\pi x)\sin (\pi y) - \cos (\pi x)\cos (\pi y) \right) \begin{bmatrix} 1 \\ -1 \end{bmatrix}, \\ p_\text {E}(t,\varvec{x})&= -\left( \cos (\eta t)-\sin (\eta t)\right) \left( \pi x\cos (\pi y)+2\pi ^2\mu \frac{\kappa }{\mu _\text {el}}\sin (\pi y)\right) , \\ \varvec{u}(t,\varvec{x})&= 2\cos (\eta t) \pi \frac{\kappa }{\mu _\text {el}} \left( \sin (\pi x)\sin (\pi y) - \cos (\pi x)\cos (\pi y) \right) \begin{bmatrix} -1 \\ 1 \end{bmatrix}, \\ p(t,\varvec{x})&= -\left( 1.5\cos (\eta t)-0.5\sin (\eta t)\right) \left( x\cos (\pi y)+4\pi ^2\mu \frac{\kappa }{\mu _\text {el}}\sin (\pi y)\right) ,\end{aligned}$$

with $\eta = \frac{\mu _\textrm{el}}{\mu _\textrm{f}(1-\alpha )}$. In particular, we enforce fully Dirichlet boundary conditions on $\partial \varOmega$, namely $\varGamma _{\text{ N },\varvec{d}}=\varGamma _{\text{ N },J}=\varGamma _{\text{ N },\varvec{u}}=\varnothing$. We simulate the system for $T=5 \cdot 10^{-7}{\hbox {s}}$ with $\varDelta t= 10^{-7}{\hbox {s}}$ and choose $s=1$ in the finite element spaces (5), namely quadratic elements for $\varvec{d},\varvec{u},p_\text {E}$ and linear elements for the Stokes pressure *p*: this choice ensures that the properties of Proposition 3.1 hold true. We set all physical parameters equal to 1, except for $\alpha _\textrm{E}=0.5$.

In the following, we discuss the results for the error energy norm24which includes all the physically relevant terms of (22). Thanks to the smoothness of the exact solution and the data employed in this test, we can safely assume that the data oscillation term  does not affect the performance of the a posteriori estimator, thus we neglect it.

**Fig. 2:**
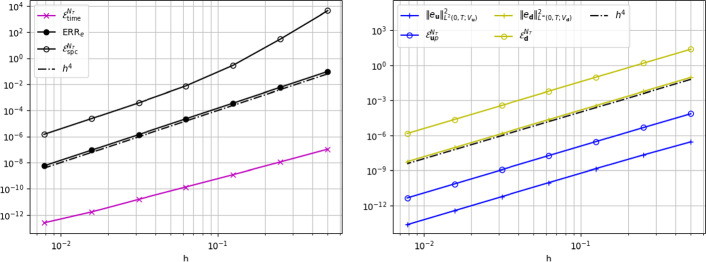
Convergence test w.r.t. space discretization parameter *h*. Left: energy error norm ${\text {ERR}_e}$ and estimators . Right: terms in the definitions of ${\text {ERR}_e}$ and  (see (24) and Theorem 3.2)

In Fig. 2 (left) we report the results of a convergence test for the error norms (24) w.r.t. *h* and the corresponding values of the estimator . The estimate (22) of Theorem 3.2 is verified by observing that  for all values of *h*. Moreover, we notice the significantly small values obtained for the estimator , due to the choice of a small value for $\varDelta t$. In Fig. 2 (right) we analyze the contribution of the different terms entering in the space error estimator . We notice that ${\mathcal {E}}_{\varvec{u}p}^{N_T}$ and ${\mathcal {E}}_{\varvec{d}}^{N_T}$ seem to be reliable estimators of  and , respectively.

**Fig. 3:**
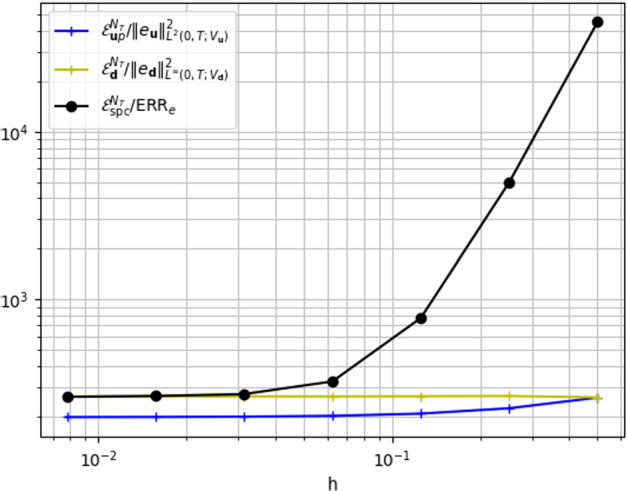
Efficiency indexes for the estimator  and of its constituting terms, in the convergence test w.r.t. *h*

To analyze the efficiency of the estimator, in Fig. 3 we display the efficiency index  and for completeness, even though not covered by our theory, we also report the analogous ratios for the single components of the error and estimator discussed above. We notice that $I_\text {eff}$ tends to a constant value for small *h*, thus meaning that the estimator is not only an upper bound of the error but rather proportional to it, thus it is also efficient. Similarly, ${\mathcal {E}}_{\varvec{u}p}^{N_T}$ and ${\mathcal {E}}_{\varvec{d}}^{N_T}$ appear to be efficient estimators of the Stokes and elastic displacement errors, respectively.

For completeness, we point out that the estimators derived here are not efficient in terms of time discretization. Specifically, by time-convergence tests not reported here for brevity, we observed that  converges w.r.t. time discretization, but  does not. This is fully consistent with the results of [16, Table 2] – in the case of the sole poroelastic problem – and of [17, Table 4] – for a simpler elliptic-parabolic problem. The study of an a-posteriori estimator suitable to time step adaptivity requires further investigation and will be the object of future research.

## Conclusions

In the present study, we have rigorously derived – for the first time – residual-based a posteriori error estimates for the finite element discretization of the coupled Stokes-MPE system. The associated estimator controls both the error in the energy norm of the system and additional terms related to the strong residual of the equations, in dual norms. We have verified the reliability and efficiency of the estimator by means of numerical experiments in a case with a manufactured solution.

The present work represents a first step towards the design of adaptive refinement algorithms and reduced order models for the efficient solution of fluid-poromechanics problems. This is particularly relevant in the study of brain multiphysics flow, in which the complexity of the geometry and fluid-tissue interface may hinder the feasibility of numerical simulations under limited resources, and in multi-query problems like model calibration and validation against clinical data [7, 31]. Further extensions of the analysis presented in this work may also be considered in the case of advanced numerical methods based on general mesh elements, such as polytopal discontinuous Galerkin and virtual element methods, in which the geometrical flexibility of the numerical scheme makes it particularly suitable for local refinement strategies [4, 9, 10, 12, 13, 18]. Moreover, a possible direction of investigation would be towards deriving reliable and efficient estimators also with respect to time discretization.

## Data Availability

Enquiries about data availability should be directed to the authors.
